# Postpandemic After-School Activities Among Youths in Australia

**DOI:** 10.1001/jamanetworkopen.2025.43637

**Published:** 2025-11-14

**Authors:** Mi Zhou, Carol Maher, Sally Brinkman, Juliette Cools, Dorothea Dumuid

**Affiliations:** 1 Alliance for Research in Exercise, Nutrition and Activity, Allied Health and Human Performance, University of South Australia, Adelaide, Australia; 2 Education Futures, University of South Australia, Adelaide, Australia; 3 South Australia Department for Education, Adelaide, Australia

## Abstract

**Importance:**

The long-term impacts of the COVID-19 pandemic on after-school activity patterns among youths remain poorly understood, hindering the development of policies to promote beneficial engagement.

**Objective:**

To examine longitudinal changes in after-school activity participation before (2019), during (2020 to 2021), and after (2022) the COVID-19 pandemic and assess whether these changes differed by sex.

**Design, Setting, and Participants:**

This longitudinal cohort study used linked yearly census survey data from the Well-Being and Engagement Collection (WEC) survey in South Australia. Participants were youths starting year 6 of school on January 29, 2019, who progressed to starting year 9 by January 31, 2022. Data analyzed were collected in weeks 2 to 5 of term 2 (May 2019 to May 2022).

**Exposure:**

COVID-19 pandemic.

**Main Outcomes and Measures:**

Weekly frequency of engagement in 11 after-school activities was measured in the WEC survey. Ordinal logistic regression models examined the trajectory of after-school activity participation. The results were visualized as estimated proportions over time.

**Results:**

A total of 14 350 participants (mean [SD] age in 2019, 11.7 [0.5] years; 7232 [50.4%] male) contributing 36 572 observations were included in the analysis. Participation in music, art, reading for fun, hanging out with friends, tutoring, and sports declined during and after the pandemic. Among these participants, youths who never participated in art (2019, 25.7% [95% CI, 24.5%-27.0%]; 2022, 70.4% [95% CI, 68.8%-72.0%]) and reading for fun (2019, 10.8% [95% CI, 10.1%-11.6%]; 2022, 52.6% [95% CI, 50.5%-54.8%]) showed the largest changes. Conversely, time spent watching television, doing chores, and playing electronic games showed meaningful increases but returned to prepandemic levels in 2022. Social media was the only activity that showed consistent increases during the 4 years, demonstrating the greatest growth. Everyday use increased from 26.0% (95% CI, 24.6%-27.3%) in 2019 to 85.4% (95% CI, 84.1%-86.7%) in 2022. Females spent more time on social media than males, while males experienced a more pronounced decline in reading.

**Conclusions and Relevance:**

This cohort study found associations between the COVID-19 pandemic and after-school activity participation patterns among youths, with rapid increases in social media use compared with all other activities. These associations largely persisted 3 years after the onset of the pandemic. These findings provide a timely benchmark for evaluating forthcoming policies aimed at restricting youth access to social media platforms. Interventions to support activities such as sports, art, and music are warranted.

## Key Points

**Question:**

How did after-school activity participation among youths change after the COVID-19 pandemic?

**Findings:**

This cohort study of 14 350 participants in South Australia during the 4 years before, during, and after the pandemic found rapid increases in social media use compared with all other activities. Everyday use of social media increased from 26% in 2019 to 85% in 2022.

**Meaning:**

These findings provide a timely benchmark for evaluating forthcoming policies aimed at restricting youths in South Australia from accessing social media platforms and suggest that interventions to support activities such as sports, art, and music are warranted.

## Teaser

This cohort study examines changes in after-school activity patterns among youths during and after the COVID-19 pandemic and whether these patterns differ by sex.

## Introduction

Youths in primary and secondary school in Australia typically have 4 to 5 hours of after-school time on school days, representing a substantial portion of their daily routine.^1^ How youths use their after-school time plays a crucial role in their development. Participation in sports, music, or adult-supervised clubs is generally considered beneficial, frequently associated with improved academic outcomes, stronger self-identity, enhanced social skills, and better mental health.^2,3,4,5,6^ These activities provide opportunities to acquire new skills, form friendships, and explore interests, thereby supporting mental and social development.^7,8,9,10,11,12^ In contrast, participation in unsupervised play, social media use, or television viewing may pose developmental risks.^2^ When screen-based leisure dominates after-school hours, youths typically engage in less physical activity and experience poorer mental health and well-being.^13,14^

Studies have documented how youths spent their after-school time before the pandemic, noting that one-third to one-half of the time was unstructured and sedentary.^15,16^ This pattern likely intensified during the COVID-19 pandemic. School closures and social distancing measures disrupted most after-school activities. Sports, clubs, and enrichment programs were widely suspended.^17^ These changes led to marked increases in screen time activities^18,19^ and decreases in sports engagement, outdoor play, and peer interaction.^20,21,22,23^

According to life course theory, major historical events such as the COVID-19 pandemic can serve as turning points that reshape life trajectories.^24,25^ The pandemic may have caused lasting shifts in youths’ after-school activity patterns. Whether youths have fully returned to prepandemic routines or whether new preferences persist, including increased reliance on digital entertainment and reduced in-person interaction, remains unclear. Longitudinal research examining how these patterns have evolved after the pandemic is lacking. This gap limits our understanding of COVID-19’s long-term impact and presents challenges for policymaking aimed at promoting youth participation in beneficial after-school activities.

Additionally, postpandemic after-school activity patterns may vary by sex, as there are known significant sex differences in activity engagement. For example, male youths tend to participate more in sports, while female youths are more active on social media.^26,27^ This disparity may have become more pronounced during the pandemic. However, few studies have explored the moderating role of sex.

This study aimed to address these gaps by examining changes in after-school activity patterns among youths during and after the COVID-19 pandemic. We investigated longitudinal trajectories in after-school activity participation and whether these patterns differed by sex.

## Methods

### Study Design and Participants

The Well-Being and Engagement Collection (WEC) is a state-wide census designed to collect data on nonacademic factors that are pertinent to the well-being of students in school years 4 to 12 in South Australia. The WEC was derived from the Canadian Middle Years Development Instrument, which has been adapted for use in Australia.^28^ The census is conducted by the South Australian Department for Education (DfE) annually in the first school term. All schools in South Australia are invited to participate. The DfE assigns participants unique identifiers, enabling the linkage of individual-level longitudinal data across time points. This cohort study received an ethics exemption from the Human Research Ethics Committee of the University of South Australia. The WEC census used a parental opt-out consent process to optimize participation rates and data representativeness. Participants were the cohort of youths in year 6 (approximately 11 years of age) in 2019 who progressed to year 9 (approximately 14 years of age) by 2022. The results of this study were reported in accordance with the Strengthening the Reporting of Observational Studies in Epidemiology (STROBE) reporting guideline.

### After-School Activity Frequency Measures

Eleven after-school activities were assessed in the WEC. The activities recorded included music lessons o practice, social media use, watching television, participation in youth organizations (eg, Scouts or Girl Guides, arts, chores, reading for fun, electronic games, hanging out with friends, homework or tutoring, and organized sports. Participants were asked to report the number of days (never, once a week, twice a week, 3 times a week, 4 times a week, or 5 times a week) they participated in each activity during the after-school period. These responses were subsequently recoded into 3 categories: none (0 d/wk), low to moderate (1-4 d/wk), and high (5 d/wk) use. The after-school activity questionnaire and distribution of after-school activities by the original 6-level frequency categories are provided in eTables 1 and 2 in Supplement 1, respectively.

### Exposure

WEC data were collected in May of each year, thus calendar year (i,e, 2019, 2020, 2021, 2022) was considered the exposure variable. The prepandemic period was captured in the May 2019 wave. The pandemic period was captured in the May 2020 and May 2021 waves, when the outbreak was as its most severe, schools underwent intermittent lockdowns, and youths faced the strictest social distancing measures. The postpandemic period was captured in the May 2022 wave, after social distancing efforts had ceased.

### Covariates

Sociodemographic information, including gender, community socioeconomic status (SES), primary language spoken at home, region of residence, and highest parental educational level, was obtained from school enrollment data reported by parents or caregivers prior to the start of each academic year. The information was administratively linked with WEC data by the DfE.

Participants reporting a gender other than male or female (alternative response options were only available in 2020) or reporting different genders across collection waves were excluded because the data were not consistently available across waves. Owing to these exclusions, our findings are reported by sex rather than by gender. Community-level SES was determined by linking each participant’s postal code to the corresponding area’s Index of Relative Socioeconomic Disadvantage (IRSD).^29^ The IRSD summarizes the economic and social conditions of a postal area by capturing relative disadvantage, with lower scores indicating greater disadvantage and higher scores indicating less disadvantage.^29^ The IRSD decile score ranged from 1 to 10 and was recoded into 3 levels for this study: 1 to 3 (low SES), 4 to 6 (medium SES), and 7 to 10 (high SES).

The primary language spoken at home was categorized as English or language other than English. The highest parental educational level referred to the highest educational attainment by either parent and was classified into 3 categories: year 12 or less (including no school qualifications, year 9 or less, year 10, year 11, and year 12), diploma (certificate I to IV, advanced diploma, or diploma), or bachelor’s degree or above. Region of residence was classified based on the Australian Bureau of Statistics Accessibility and Remoteness Index of Australia into major city, inner and outer regional, and remote and very remote regional.^30^

### Statistical Analysis

Data were analyzed from May 2019 to May 2022. Ordinal logistic regression models were used with calendar year as the independent variable and frequency of after-school activities as dependent variables. Participant’s sex, age, residential region, highest parental educational level, primary language spoken at home, and community SES were included as covariates. School and individual levels were considered as independent random intercepts to account for clustering of participants within schools and repeated observations within individuals. This process was repeated for the interaction models (year × sex). Adjusted estimated proportions of frequency for each activity at each calendar year were calculated and plotted to facilitate visual comparisons. All analyses were conducted in R statistical software, version 4.3.1 (R Program for Statistical Computing). The ordinal logistic regression model was built with the ordinal package, estimates were calculated with ggeffects, and ggplot was used to visualize the results.^31,32,33^

## Results

### Participant Characteristics

A total of 14 350 participants were included in this study, contributing 36 572 observations during the 4-year study period (eTable 3 in Supplement 1). On average, each individual student completed 2.5 annual surveys. A participant flowchart is provided in the eFigure in Supplement 1. A total of 4181 students were excluded, with excluded characteristics reported in eTable 3 in Supplement 1. Baseline characteristics were comparable between included and excluded participants.

The included sample exhibited a roughly equal distribution of sex, with 4888 (49.6%) female and 4963 (50.4%) male participants at baseline. English was the most common language spoken at home (ranging from 5569 of 6975 [79.8%] in 2022 to 7099 of 8664 [81.9%] in 2019). Most participants (ranging from 5430 of 7862 [69.1%] in 2022 to 7057 of 9846 [71.7%] in 2019) resided in major cities, while only a small proportion lived in remote or very remote areas (ranging from 278 of 9846 [2.8%] in 2019 to 355 of 9485 [3.7%] in 2021). Slightly less than one-half of parents had a diploma-level education (ranging from 4415 of 9450 [46.7%] in 2019 to 3649 of 7654 [47.7%] in 2022), and approximately one-third (ranging from 3040 of 9046 [33.6%] in 2020 to 2715 of 7654 [35.5%] in 2022) had a bachelor’s degree or higher qualification level.

### After-School Activity Frequency Changes Across 4 Years

**Figure 1.**
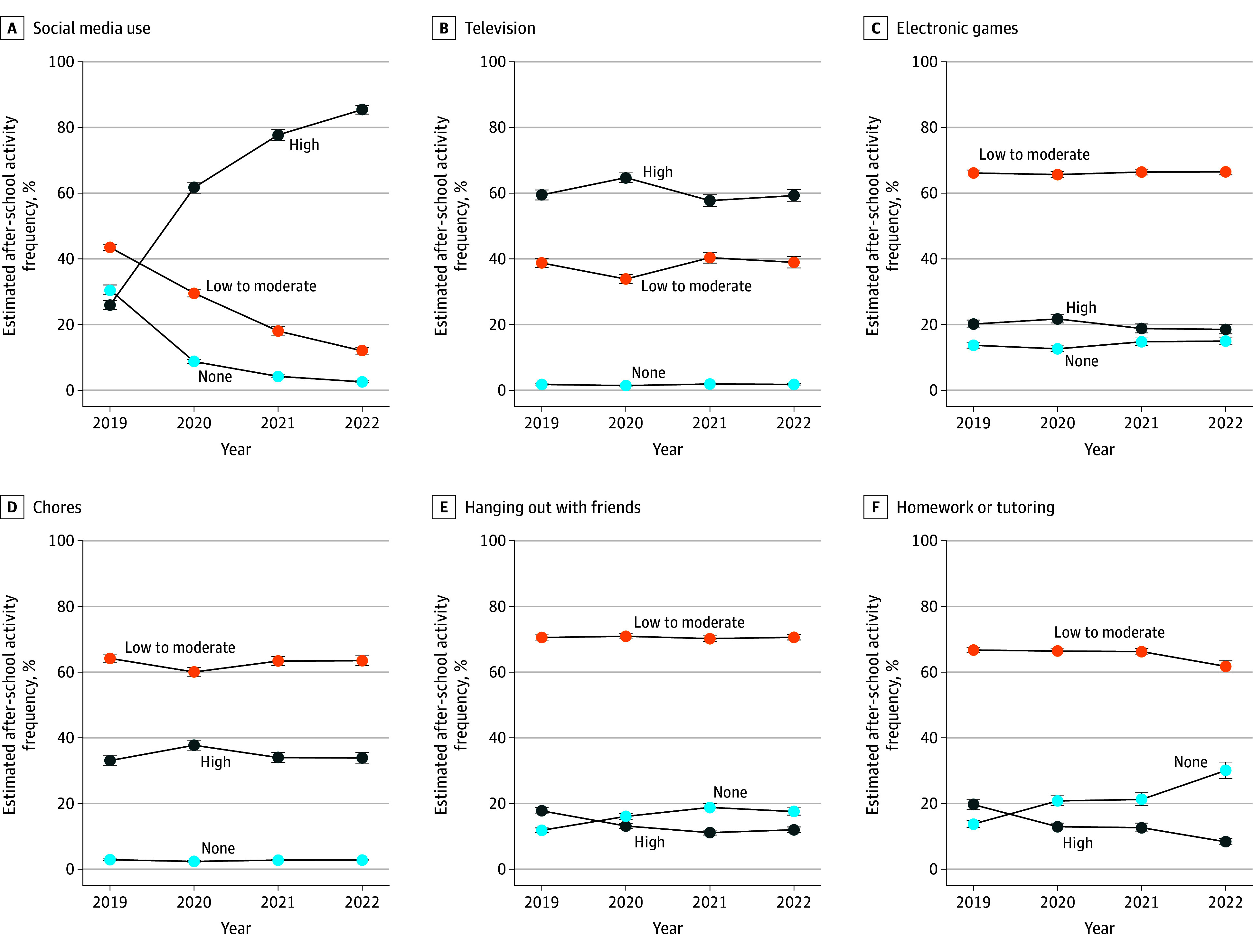
Frequency of After-School Participation in Social Media Use, Television, Electronic Games, Chores, Hanging Out With Friends, and Homework or Tutoring None indicates never use; high, 5 d/wk.

**Figure 2.**
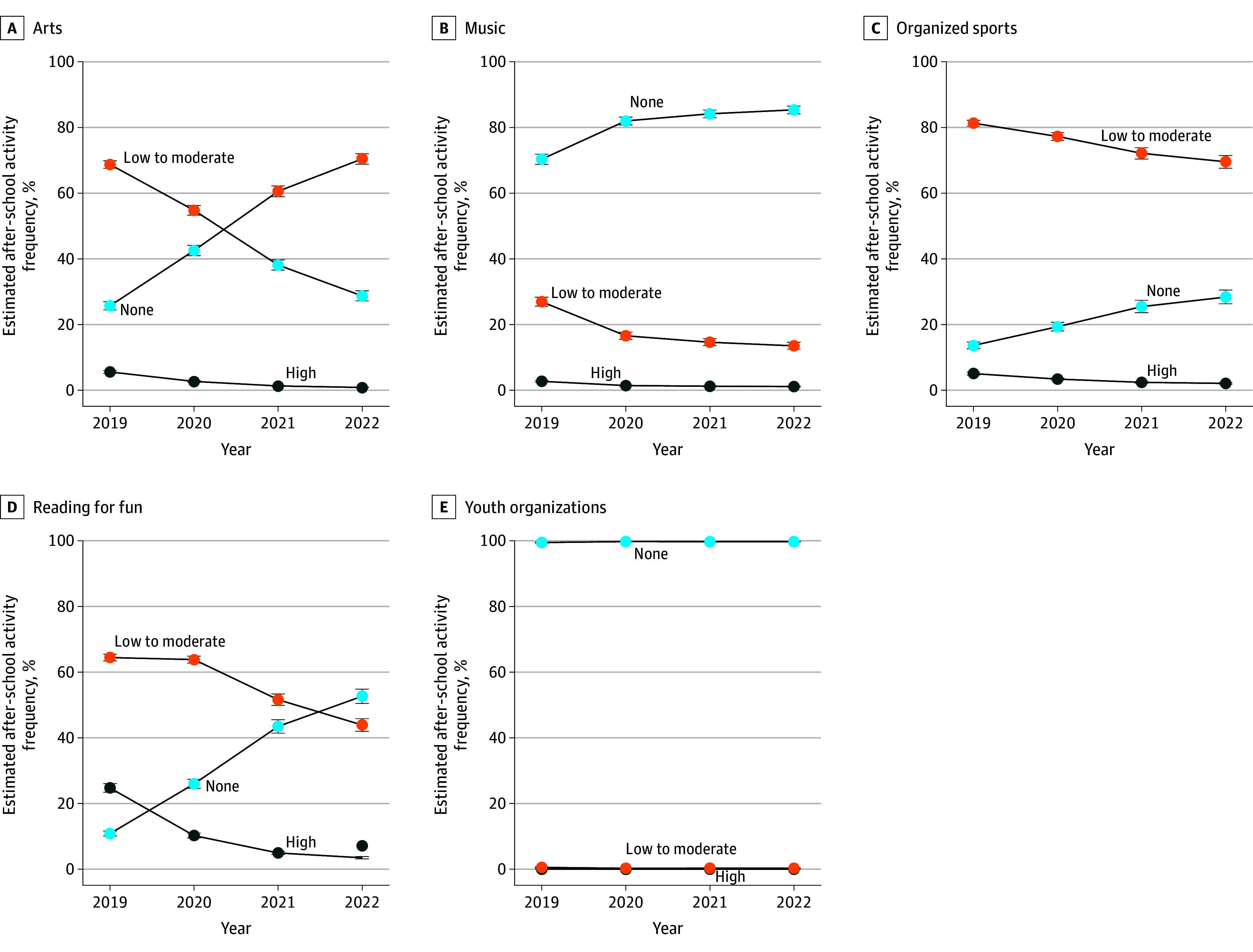
Frequency of After-School Participation in Arts, Music, Organized Sports, Reading, and Youth Organizations None indicates never use; high, 5 d/wk.

Figure 1 and Figure 2 show the estimated proportions of after-school activity frequency from 2019 to 2022. Social media use increased markedly, with daily use (5 d/wk) rising from 26.0% (95% CI, 24.6%-27.3%) in 2019 to 85.4% (95% CI, 84.1%-86.7%) in 2022, while never use decreased from 30.6% (95% CI, 29.1%-32.1%) in 2019 to 2.6% (95% CI, 2.3%-2.9%) in 2022 (Figure 1 and eTable 4 in Supplement 1). Participation in watching television, electronic games, and chores remained relatively stable. These activities showed a temporary increase in the proportion of use 5 d/wk, but returned to prepandemic levels within 2 years (Figure 1).

Arts and reading for fun saw the greatest declines (Figure 2). The proportion of youths who never participated in arts increased from 25.7% (95% CI, 24.5%-27.0%) in 2019 to 70.4% (95% CI, 68.8%-72.0%) in 2022, and those who never read for fun increased from 10.8% (95% CI, 10.1%-11.6%) in 2019 to 52.6% (95% CI, 50.5%-54.8%). Never participating in extracurricular music activities increased from 70.3% (95% CI, 68.8%-71.9%) in 2019 to 85.4% (95% CI, 84.1%-86.5%) in 2020. Low to moderate participation also declined from 68.7% (95% CI, 67.6%-69.9%) in 2019 to 28.7% (95% CI, 27.2%-30.3%) in 2022 in arts and from 64.4% (95% CI, 63.4%-65.4%) in 2019 to 43.9% (95% CI, 42.0%-45.8%) in 2022 in reading (eTable 4 in Supplement 1). Youth organization involvement remained consistently low, with approximately 99% of respondents reporting no participation throughout the 4-year period (Figure 2).

### After-School Activity Frequency Changes During 4 Years Across Sex

**Figure 3.**
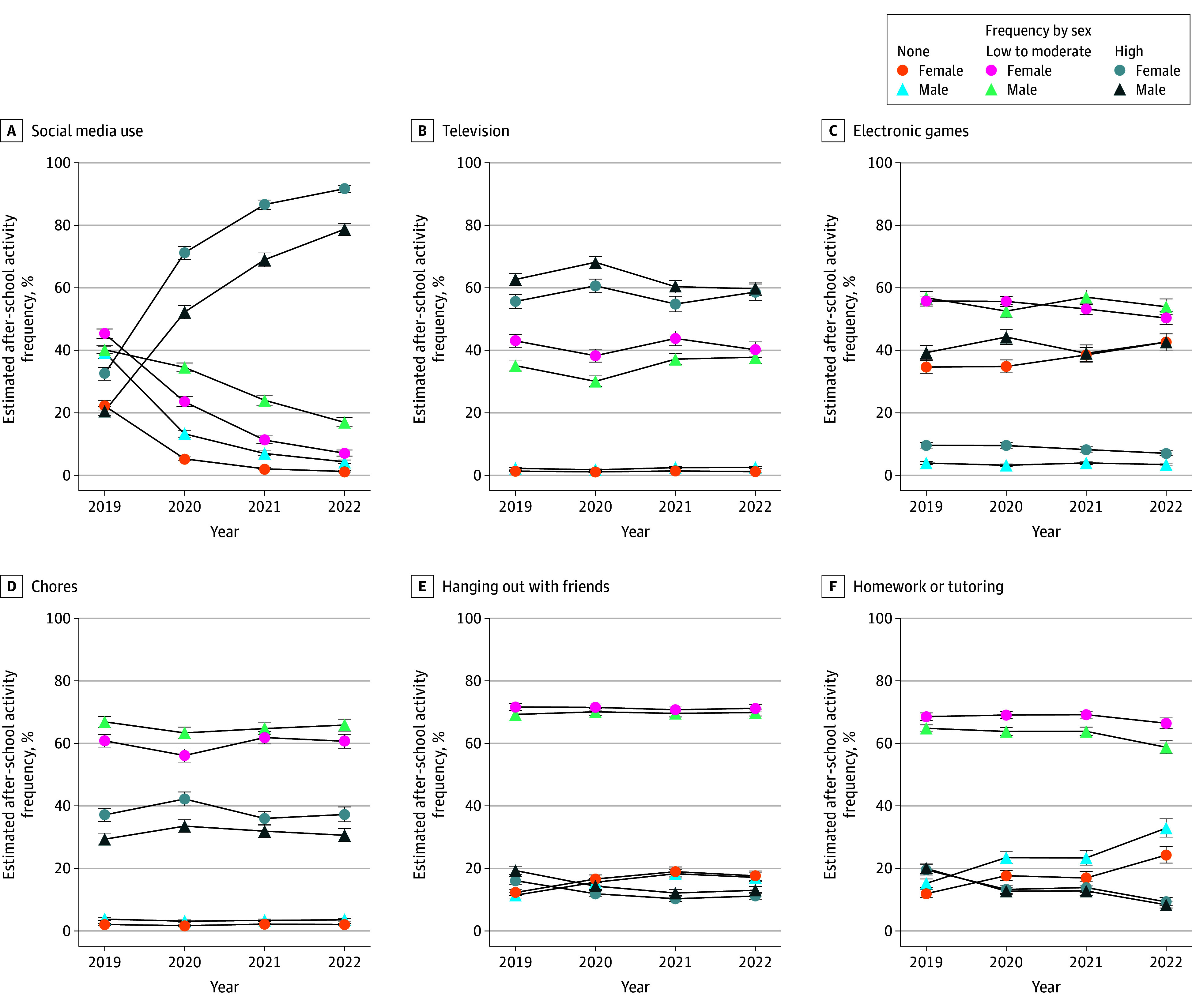
Frequency of After-School Participation in Social Media Use, Television, Electronic Games, Chores, Hanging Out With Friends, and Homework or Tutoring by Sex None indicates never use; high, 5 d/wk.

Over time, the proportion of females engaging in social media at a frequency of 5 d/wk increased from 32.4% (95% CI, 30.4%-34.5%) in 2019 to 91.7% (95% CI, 90.5%-92.8%) in 2022, whereas use among males rose from 20.5% (95% CI, 19.1%-22.0%) to 78.7% (95% CI, 76.7%-80.6%) during the same period (Figure 3 and eTable 5 in Supplement 1). Concurrently, the proportion of respondents never using social media significantly decreased for both sexes from 22.3% (95% CI, 20.6%-24.0%) in 2019 to 1.2% (95% CI, 1.0%-1.5%) in 2022 for females and from 39.3% (95% CI, 37.3%-41.3%) in 2019 to 4.3% (95% CI, 3.8%-4.9%) in 2022 for males (Figure 3 and eTable 5 in Supplement 1).

**Figure 4.**
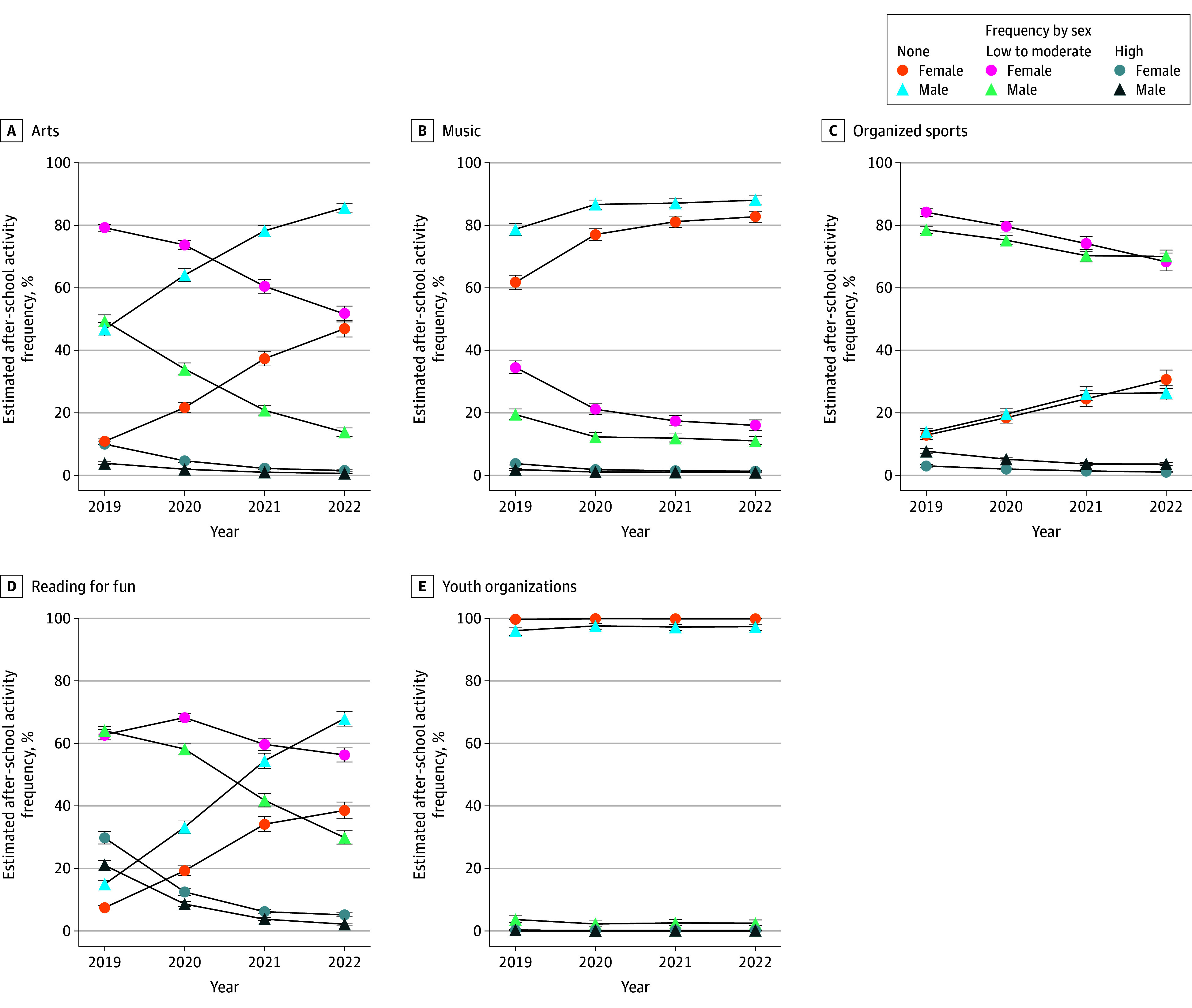
Frequency of After-School Participation in Arts, Music, Organized Sports, Reading, and Youth Organizations by Sex None indicates never use; high, 5 d/wk.

The proportion of females engaging in reading 5 d/wk was notably higher than that of males in 2019 (29.8% [95% CI, 27.8%-31.8%] vs 21.0% [95% CI, 19.5%-22.6%]) (Figure 4 and eTable 5 in Supplement 1). However, by 2022, the proportions for both sexes had decreased to similarly low levels (female, 5.1% [95% CI, 4.5%-5.8%]; male, 2.2% [95% CI, 1.9%-2.5%]). In contrast, the proportion of males who never engaged in reading rose dramatically from 15.0% (95% CI, 13.8%-16.2%) in 2019 to 68.0% (95% CI, 65.6%-70.3%) in 2022. Females experienced a similar but less pronounced increase, from 7.4% (95% CI, 6.7%-8.2%) to 38.6% (95% CI, 35.9%-41.3%) (Figure 4 and eTable 5 in Supplement 1).

## Discussion

### Main Findings

In this cohort study, we found that participation in music, art, reading for fun, hanging out with friends, tutoring, and sports declined during and after the COVID-19 pandemic. Conversely, time spent watching television, doing chores, and playing electronic games increased significantly but returned to prepandemic levels in the subsequent 2 years. Social media was the only activity that consistently increased throughout the pandemic and postpandemic years, showing the largest growth. Females spent more time on social media than males, while males experienced a more pronounced decline in reading.

### Comparisons With Previous Research

The unprecedented rise in social media observed during the pandemic has been reported.^34,35,36,37,38^ Our findings are consistent with this research. Only a few studies using longitudinal research^36,39^ have reported on how youths’ social media use changed after the pandemic. One report^39^ indicated that children’s screen time increased during the pandemic but declined after restrictions were lifted, although remaining higher than prepandemic levels. Another study^36^ indicated that youths’ social media use time remained high after public health measures were lifted. These studies collectively indicate that social media behavior patterns among youths shifted after the pandemic. Our results extend 1 year beyond the aforementioned studies, finding that social media use among youths maintains an upward trend 3 years after the onset of the pandemic.

Current research on whether sex moderates the association between the pandemic and changes in social media use presents mixed findings. Two recent systematic reviews^40,41^ reported no clear sex moderation effect on daily screen time changes from before to after the pandemic. Similarly, a recent longitudinal cohort study that followed up 228 children aged 4 to 12 years at 3 time points before and during the COVID-19 pandemic^36^ found no moderating effect of sex on changes in social media use. Our study indicated that female youths consistently reported higher levels of social media use than male youths during and after the pandemic, aligning with a portion of the literature.^42^

In line with our findings, a 3-year longitudinal study of after-school screen time during and after the pandemic among youths in Israel^35^ showed that time watching television and playing video games increased during the pandemic. However, in contrast with our study, which suggested the effect subsided after the pandemic, they reported continuing high levels of watching television and playing videogames.^35^ One possible explanation is that Australian parents implement strict supervision of traditional screen entertainment activities. Research shows that approximately 70% of Australian parents set limits on their child’s weekly screen time.^43^ Another explanation is that the rise of social media has displaced time youths originally spent on traditional screen activities (such as television and video games). Considering youths’ limited screen time, increased social media preference may have reduced their engagement with television and video games. This explanation should be validated by further research.

Contrary to our findings, 1 study^44^ reported that youths’ investment in music activities increased during the pandemic, with many caregivers participating in music activities with their children to cope with pandemic-related psychological stress. In our study, the proportion of youths who originally never participated in extracurricular music and art programs drastically increased from 70.3% and 25.7% in 2019 to 85.4% and 70.4% in 2020, which may indicate that the pandemic prompted them to actively or passively terminate these activities earlier. However, this transition typically occurs during the process of transitioning from adolescence to adulthood.^45^ The long-term impact of this premature withdrawal remains unclear and requires further research.

Previous studies have reported a decline in youth participation in reading for fun during the pandemic, which aligns with our findings.^46,47,48^ Our study found this decline has not returned to prepandemic levels even after restrictions were lifted. We also revealed significant sex differences, whereas no previous research has reported such sex disparities during and after the pandemic.

Similarly, the frequency of youth participation in sports declined during the pandemic.^49^ The latest research shows that physical activity duration among youths decreased during the pandemic and returned to prepandemic levels once restrictions were lifted.^50^ However, the time spent in school sports clubs remains low, which is consistent with our findings.^50^ Current evidence on sex-moderating effects is mixed.^49^ Our study found minimal differences between males and females in sports participation during the pandemic, which aligns with findings from several other studies.^50,51,52^

### Interpretations and Implications

Due to home isolation measures, restrictions on face-to-face social activities, and the widespread adoption of remote learning, social media use surged among youths during the pandemic.^34,35,36^ The rise of social media, to the detriment of other after-school activities, is problematic given the weight of evidence linking higher social media use with poor mental health, poor sleep quality, and obesity.^53,54,55^ However, social media use also has benefits.^56^ For instance, social media could help maintain connections with family members and peers during lockdown and serve as a tool to alleviate pandemic-related stress.^57^ These findings underscore the double-edged sword represented by social media, suggesting that its impacts are highly context dependent and are shaped by how, why, and under what circumstances it is used.

Moreover, the impact of social media depends not merely on frequency or duration of use, but also on patterns of use, content type, and users’ individual characteristics.^34,58^ A typical example is the difference in social media patterns of use between sexes. Taking our results as an example, female youths consistently reported higher levels of social media use than male youths during the pandemic. Possible reasons include their greater concern about pandemic developments, experiencing more intense psychological distress, and using social media as a means of emotional connection and to alleviate anxiety and loneliness.^59,60,61^ Under such conditions, even increased use might be beneficial. In contrast, male youths tend more toward passive social media use for entertainment purposes rather than social interaction.^58,62^ This passive media use pattern is associated with decreased well-being.^58,63^ Therefore, interpretations of the rise in social media use and its impacts should be cautious and context sensitive, rather than relying on a label of excessive use as a uniform criterion.

Another pattern revealed by this study is the continuous decline in leisure reading among youths that extended into the postpandemic period. As of 2022, the proportion of youths reporting never reading in a week has exceeded the combined proportion of those who report reading occasionally and reading daily, signifying a meaningful shift in youth reading habits before and after the pandemic. Notably, the downward movement is more pronounced among male youth.

Research has confirmed a positive correlation between intrinsic reading motivation and reading behavior. Youths who read for interest and enjoyment have significantly higher reading frequency than youths who read due to external motivation (such as avoiding punishment or obtaining rewards).^64,65,66,67^ In educational contexts, the contribution of intrinsic motivation to reading frequency is stronger for leisure reading, while relatively weaker for academic reading.^64^ During the pandemic, although the time invested by youths in academic tasks increased, their concentration significantly decreased,^68^ reflecting that their engagement in academic activities was more passive. In this study, changes in homework engagement were minimal, while leisure reading showed a meaningful decline. This perhaps suggests that intrinsic reading motivation is experiencing rapid and sustained weakening among youths.

This pattern is similarly reflected in broader after-school activities. Activities typically considered driven by intrinsic motivation, such as music, art, and sports, showed generally declining participation rates during the 4 years of the study; activities such as household chores, more easily driven by external motivation, showed no significant changes. This may indicate that youths’ after-school activity participation patterns are undergoing a systematic shift from internally driven to externally driven. Such a shift could have important implications for long-term development and well-being, potentially limiting opportunities for creativity, autonomy, and personal growth.

### Suggestions for Future Research

This research suggests several avenues for future research. First, this study identified changes in youths’ after-school activity patterns before and after the pandemic. However, the impact of these changes on youth health and well-being remains unclear. Second, as participants were transitioning from childhood to adolescence, a critical developmental period, these behavioral shifts may have lasting effects. Given that the study only examined activities to 3 years after the onset of the pandemic, future research should explore how these patterns evolve across the life course. Additionally, this study focused solely on sex differences in after-school activity patterns. Structured programs such as music and arts often involve participation fees, which can limit access for low-income families. Youths from higher-income or more educated households are more likely to engage in these activities.^69,70,71^ These disparities may have been exacerbated during and after the pandemic. Further research is warranted to explore after-school activity patterns across different socioeconomic statuses and demographic groups.

Finally, although television and gaming habits did not show significant changes during the pandemic, they warrant consideration nonetheless. On November 29, 2024, the Australian federal government amended the Online Safety Act to prohibit children younger than 16 years from holding social media accounts, currently the most stringent social media ban in the world.^72^ When access to social media is restricted, children may shift to alternative platforms such as gaming, messaging apps, or video websites, which are not covered by the ban.^72^ Their television and gaming times may show an upward trend in the coming years. It remains essential to pay close attention to youth engagement across various forms of screen-based activities.

### Strengths and Limitations

To the best of our knowledge, this study represents the largest global longitudinal research to date on after-school activities. This study includes a large sample size with broad representation across geographic regions and socioeconomic backgrounds, as well as the ability to link individual participants over time. The longitudinal design enables the analysis of behavioral changes spanning both the prepandemic and postpandemic periods and provides valuable insights into how major global disruptions may influence children’s and adolescents’ daily routines and lifestyle habits.

This study also has some limitations. We only reported the frequency of youth participation in after-school activities, not the actual time (duration) spent on each activity; therefore, it may not accurately reflect time allocation across different fields. Additionally, the longitudinal cohort design of this study means we cannot disentangle developmental-related changes in activities from temporal changes. Furthermore, this study relied on self-reported questionnaire data, which may be subject to recall bias and social desirability effects. More objective measurement methods could be considered in the future. Finally, since the sample comes from South Australia, caution should be exercised when generalizing these findings to other regions of the world.

## Conclusions

The findings of this cohort study suggest that the COVID-19 pandemic had important associations with the after-school activity participation patterns of youths in South Australia, with rapid increases in social media use compared with all other activities. Participation in music, art, reading for fun, hanging out with friends, tutoring, and sports declined during and after the pandemic. Conversely, time spent on television, chores, and electronic games increased significantly but returned to prepandemic levels in the subsequent 2 years. Females spent more time on social media than males, while males experienced a more pronounced decline in reading. These findings provide a benchmark for assessing the impact of an impending policy change that bans social media for children and adolescents in South Australia. Interventions to support activities such as sports, art, and music are warranted. Future research should further explore the impact of postpandemic after-school activity patterns on youth health and well-being outcomes.

## Notes

Supplement 1. **eTable 1.** After-School Activity Questionnaire **eTable 2.** Distribution of After-School Activities by the Original 6-Level Frequency Categories **eTable 3.** Characteristics of Participants and After-School Activity Frequency **eTable 4.** Estimated Proportions in After-School Activity Frequency for Each Activity From 2019 to 2022 **eTable 5.** Estimated Proportions in After-School Activity Frequency for Each Activity by Sex From 2019 to 2022 **eFigure.** Participant Flowchart

Supplement 2.
Data Sharing Statement
