# Supplementary material for: Postpandemic After-School Activities Among Youths in Australia
Source: JAMA Netw Open. 2025 Nov 14;8(11):e2543637. doi: 10.1001/jamanetworkopen.2025.43637 (PMC12619106; doi:10.1001/jamanetworkopen.2025.43637)
Supplement: Supplement 1. — eTable 1. After-School Activity Questionnaire eTable 2. Distribution of After-School Activities by the Original 6-Level Frequency Categories eTable 3. Characteristics of Participants and After-School Activity Frequency eTable 4. Estimated Proportions in After-School Activity Frequency for Each Activity From 2019 to 2022 eTable 5. Estimated Proportions in After-School Activity Frequency for Each Activity by Sex From 2019 to 2022 eFigure. Participant Flowchart [file jamanetwopen-e2543637-s001.pdf]

Supplementary Online Content

Zhou M, Maher C, Brinkman S, Cools J, Dumuid D. Postpandemic after-school activities among youths in Australia. *JAMA Netw Open.* 2025;8(11):e2543637. doi:10.1001/jamanetworkopen.2025.43637

eTable 1. After-School Activity Questionnaire
eTable 2. Distribution of After-School Activities by the Original 6-Level Frequency Categories
eTable 3. Characteristics of Participants and After-School Activity Frequency
eTable 4. Estimated Proportions in After-School Activity Frequency for Each Activity From 2019 to 2022
eTable 5. Estimated Proportions in After-School Activity Frequency for Each Activity by Sex From 2019 to 2022
eFigure. Participant Flowchart

This supplementary material has been provided by the authors to give readers additional information about their work.

© 2025 Zhou M et al. *JAMA Network Open*.

eTable 1. After-School Activity Questionnaire

No.	Code	Question Item	Answer					
			0 (Never)	1	2	3	4	5 (Every day)
**1**	Social media	In a usual week, how many days do you use a phone or the Internet to text or chat with friends and/or go on social networking sites like Facebook, Instagram or Snapchat?						
2	TV	In a usual week, how many days do you watch TV (including Netflix, Youtube or DVDs						
3	E-games	In a usual week, how many days do you play video or computer games (for example, Play Station, Xbox, multi-user online games						
4	Chores	In a usual week, how many days do you do household chores (for example, clean your room, wash the dishes, feed a pet, work on the farm or something else						
5	Hang out with friends	In a usual week, how many days do you hang out with friends?						
6	Homework/Tutor	In a usual week, how many days do you do homework, and/or participate in educational lessons or activities (for example, tutoring, maths, language school, or something else						
7	Arts	In a usual week, how many days do you do arts and crafts (including painting, drawing, or something else						
8	Music	In a usual week, how many days do you do music lessons or practice a musical instrument (for example, drums, guitar, violin, piano or something else						
9	Organized sports	In a usual week, how many days do you do organised individual or team sports (for example, basketball, swimming, cricket, football, netball, dancing, or something else						
10	Read for fun	In a usual week, how many days do you read for fun?						
11	Young organizations	In a usual week, how many days do you participate in Youth organisations (for example, Scouts, Girl Guides, Boys and Girls Clubs, or something else						

© 2025 Zhou M et al. *JAMA Network Open*.

eTable 2. Distribution of After-School Activities by the Original 6-Level Frequency Categories

After-school activity	Frequency level (days per week)	2019 (N=9851)	2020 (N=9365)	2021 (N=9488)	2022 (N=7868)
Social media	0	3408 (36.2%)	1429 (15.8%)	724 (7.9%)	433 (5.5%)
	1	803 (8.5%)	488 (5.4%)	343 (3.7%)	199 (2.5%)
	2	684 (7.3%)	491 (5.4%)	381 (4.1%)	231 (3%)
	3	740 (7.9%)	656 (7.3%)	521 (5.7%)	383 (4.9%)
	4	656 (7%)	640 (7.1%)	648 (7%)	448 (5.7%)
	5	3129 (33.2%)	5320 (59%)	6582 (71.6%)	6131 (78.4%)
TV	0	301 (3.2%)	270 (3%)	376 (4.1%)	373 (4.8%)
	1	595 (6.4%)	480 (5.3%)	564 (6.2%)	426 (5.5%)
	2	890 (9.5%)	720 (8%)	812 (8.9%)	637 (8.2%)
	3	1138 (12.1%)	1027 (11.4%)	1133 (12.4%)	1024 (13.2%)
	4	1180 (12.6%)	1037 (11.6%)	1202 (13.2%)	907 (11.7%)
	5	5266 (56.2%)	5438 (60.6%)	5053 (55.3%)	4404 (56.7%)
E-games	0	2272 (24.6%)	2123 (23.9%)	2364 (25.8%)	2139 (27.5%)
	1	1206 (13.1%)	1024 (11.5%)	1099 (12%)	867 (11.1%)
	2	1091 (11.8%)	961 (10.8%)	1033 (11.3%)	830 (10.7%)
	3	1066 (11.6%)	1083 (12.2%)	1087 (11.9%)	909 (11.7%)
	4	779 (8.4%)	755 (8.5%)	857 (9.4%)	689 (8.8%)
	5	2812 (30.5%)	2945 (33.1%)	2711 (29.6%)	2354 (30.2%)
Chores	0	782 (8.5%)	586 (6.6%)	629 (6.9%)	549 (7.1%)
	1	1200 (13%)	1021 (11.4%)	966 (10.6%)	738 (9.5%)
	2	1200 (13%)	1231 (13.8%)	1300 (14.2%)	1108 (14.3%)
	3	1468 (15.9%)	1443 (16.1%)	1692 (18.5%)	1398 (18.1%)
	4	957 (10.4%)	900 (10.1%)	983 (10.7%)	860 (11.1%)
	5	3631 (39.3%)	3765 (42.1%)	3582 (39.1%)	3078 (39.8%)
Hang out with friends	0	1971 (21%)	2104 (23.4%)	2043 (22.1%)	1735 (22.3%)
	1	2121 (22.6%)	2112 (23.5%)	2233 (24.2%)	1741 (22.4%)
	2	1267 (13.5%)	1400 (15.5%)	1734 (18.8%)	1363 (17.6%)

© 2025 Zhou M et al. *JAMA Network Open*.

	3	849 (9%)	1025 (11.4%)	1246 (13.5%)	1134 (14.6%)
	4	560 (6%)	510 (5.7%)	612 (6.6%)	558 (7.2%)
	5	2628 (28%)	1854 (20.6%)	1360 (14.7%)	1235 (15.9%)
**Homework/Tutor**	0	1984 (21.1%)	2557 (28.5%)	1834 (19.7%)	2075 (26.7%)
	1	1478 (15.7%)	1469 (16.4%)	1331 (14.3%)	1365 (17.6%)
	2	1072 (11.4%)	1131 (12.6%)	1521 (16.4%)	1376 (17.7%)
	3	1123 (11.9%)	1107 (12.3%)	1813 (19.5%)	1331 (17.1%)
	4	1145 (12.2%)	904 (10.1%)	1138 (12.2%)	615 (7.9%)
	5	2601 (27.7%)	1815 (20.2%)	1660 (17.9%)	1015 (13.1%)
**Arts**	0	3224 (33.9%)	4127 (45.4%)	5254 (56.5%)	4959 (63.9%)
	1	2213 (23.3%)	2051 (22.6%)	1713 (18.4%)	1121 (14.4%)
	2	1266 (13.3%)	1019 (11.2%)	910 (9.8%)	589 (7.6%)
	3	1041 (11%)	748 (8.2%)	637 (6.9%)	457 (5.9%)
	4	495 (5.2%)	396 (4.4%)	290 (3.1%)	261 (3.4%)
	5	1261 (13.3%)	753 (8.3%)	489 (5.3%)	379 (4.9%)
**Music**	0	5878 (61.8%)	6508 (71.5%)	6812 (73.2%)	5805 (74.9%)
	1	1481 (15.6%)	1061 (11.7%)	988 (10.6%)	638 (8.2%)
	2	603 (6.3%)	456 (5%)	452 (4.9%)	356 (4.6%)
	3	474 (5%)	351 (3.9%)	365 (3.9%)	324 (4.2%)
	4	254 (2.7%)	192 (2.1%)	199 (2.1%)	202 (2.6%)
	5	826 (8.7%)	535 (5.9%)	484 (5.2%)	429 (5.5%)
**Organised sports**	0	2304 (24.4%)	2789 (30.8%)	3240 (34.6%)	2994 (38.2%)
	1	1792 (18.9%)	1517 (16.8%)	1427 (15.2%)	989 (12.6%)
	2	1833 (19.4%)	1683 (18.6%)	1797 (19.2%)	1365 (17.4%)
	3	1272 (13.4%)	1206 (13.3%)	1321 (14.1%)	1169 (14.9%)
	4	887 (9.4%)	829 (9.2%)	815 (8.7%)	633 (8.1%)
	5	1372 (14.5%)	1021 (11.3%)	760 (8.1%)	683 (8.7%)
**Read for fun**	0	1832 (19.9%)	3084 (34.4%)	4233 (47%)	4112 (53%)
	1	1416 (15.4%)	1540 (17.2%)	1496 (16.6%)	1109 (14.3%)
	2	1133 (12.3%)	1104 (12.3%)	927 (10.3%)	725 (9.3%)

© 2025 Zhou M et al. *JAMA Network Open*.

	3	1085 (11.8%)	912 (10.2%)	787 (8.7%)	614 (7.9%)
	4	819 (8.9%)	567 (6.3%)	436 (4.8%)	336 (4.3%)
	5	2933 (31.8%)	1766 (19.7%)	1123 (12.5%)	864 (11.1%)
**Young organization**	0	7998 (84.3%)	8023 (88.5%)	8164 (88%)	6850 (88.3%)
	1	775 (8.2%)	665 (7.3%)	717 (7.7%)	535 (6.9%)
	2	248 (2.6%)	148 (1.6%)	147 (1.6%)	121 (1.6%)
	3	143 (1.5%)	71 (0.8%)	85 (0.9%)	111 (1.4%)
	4	93 (1%)	35 (0.4%)	68 (0.7%)	52 (0.7%)
	5	232 (2.4%)	123 (1.4%)	98 (1.1%)	93 (1.2%)

© 2025 Zhou M et al. *JAMA Network Open*.

eTable 3. Characteristics of Participants and After-School Activity Frequency

		2019		2020		2021		2022	
**Variable name**	**Levels**	**Included (N=9851)**	**Excluded (N=898)**	**Included (N=9365)**	**Excluded (N=2076)**	**Included (N=9488)**	**Excluded (N=631)**	**Included (N=7868)**	**Excluded (N=576)**
**Gender**	Female	4888 (49.6%)	306 (47.4%)	4595 (49.1%)	1003 (48.3%)	4689 (49.4%)	228 (36.1%)	3883 (49.4%)	200 (34.7%)
	Male	4963 (50.4%)	340 (52.6%)	4770 (50.9%)	1073 (51.7%)	4799 (50.6%)	403 (63.9%)	3985 (50.6%)	376 (65.3%)
**Age**	Mean ± SD	11.7 ± 0.5	10.9 ± 0.5	12.7 ± 0.5	12.1 ± 0.5	13.7 ± 0.5	12.9 ± 0.4	14.7 ± 0.5	13.9 ± 0.7
**Main language spoken at home**	English	7099 (81.9%)	743 (82.7%)	6618 (80%)	1782 (85.8%)	6773 (80.6%)	539 (85.4%)	5569 (79.8%)	465 (80.7%)
	LOTE	1565 (18.1%)	155 (17.3%)	1655 (20%)	294 (14.2%)	1632 (19.4%)	92 (14.6%)	1406 (20.2%)	111 (19.3%)
**Regional area**	Major city	7057 (71.7%)	697 (77.7%)	6619 (70.7%)	1471 (70.9%)	6562 (69.2%)	441 (70.1%)	5430 (69.1%)	441 (76.6%)
	Inner & outer regional	2511 (25.5%)	184 (20.5%)	2406 (25.7%)	542 (26.1%)	2568 (27.1%)	172 (27.3%)	2133 (27.1%)	123 (21.4%)
	Remote & very remote regional	278 (2.8%)	16 (1.8%)	338 (3.6%)	62 (3%)	355 (3.7%)	16 (2.5%)	299 (3.8%)	12 (2.1%)
**Highest parental education**	Year 12 or less	1742 (18.4%)	138 (20%)	1689 (18.7%)	338 (17%)	1628 (17.7%)	135 (22.4%)	1290 (16.9%)	111 (19.9%)
	Diploma	4415 (46.7%)	328 (47.6%)	4317 (47.7%)	1000 (50.2%)	4383 (47.6%)	297 (49.3%)	3649 (47.7%)	277 (49.6%)
	Bachelor degree or above	3293 (34.8%)	223 (32.4%)	3040 (33.6%)	656 (32.9%)	3203 (34.8%)	171 (28.4%)	2715 (35.5%)	171 (30.6%)
**IRSD**	Low SES	3735 (38%)	327 (36.6%)	3631 (38.8%)	765 (36.9%)	3541 (37.3%)	249 (39.6%)	2827 (36.6%)	220 (38.2%)
	Mid SES	3208 (32.6%)	307 (34.3%)	3187 (34.1%)	674 (32.5%)	3217 (33.9%)	208 (33.1%)	2617 (33.9%)	188 (32.6%)
	High SES	2892 (29.4%)	260 (29.1%)	2536 (27.1%)	635 (30.6%)	2724 (28.7%)	171 (27.2%)	2283 (29.5%)	168 (29.2%)
**After-school activity frequency**									
**Social media**	None (Never)	3408 (36.2%)	149 (32.2%)	1429 (15.8%)	224 (12.2%)	724 (7.9%)	19 (10%)	433 (5.5%)	7 (4.7%)
	Low/Moderate Usage	2883 (30.6%)	173 (37.4%)	2275 (25.2%)	394 (21.5%)	1893 (20.6%)	41 (21.6%)	1261 (16.1%)	31 (20.8%)
	High (Everyday)	3129 (33.2%)	141 (30.5%)	5320 (59%)	1212 (66.2%)	6582 (71.6%)	130 (68.4%)	6131 (78.4%)	111 (74.5%)

© 2025 Zhou M et al. *JAMA Network Open*.

**TV**	None (Never)	301 (3.2%)	10 (2.2%)	270 (3%)	29 (1.6%)	376 (4.1%)	10 (5.2%)	373 (4.8%)	10 (6.8%)
	Low/Moderate Usage	3803 (40.6%)	190 (40.9%)	3264 (36.4%)	450 (24.6%)	3711 (40.6%)	72 (37.7%)	2994 (38.5%)	45 (30.6%)
	High (Everyday)	5266 (56.2%)	264 (56.9%)	5438 (60.6%)	1347 (73.8%)	5053 (55.3%)	109 (57.1%)	4404 (56.7%)	92 (62.6%)
**E-games**	None (Never)	2272 (24.6%)	108 (23.7%)	2123 (23.9%)	327 (17.9%)	2364 (25.8%)	36 (19.1%)	2139 (27.5%)	30 (20.1%)
	Low/Moderate Usage	4142 (44.9%)	199 (43.7%)	3823 (43%)	782 (42.9%)	4076 (44.5%)	84 (44.7%)	3295 (42.3%)	62 (41.6%)
	High (Everyday)	2812 (30.5%)	148 (32.5%)	2945 (33.1%)	714 (39.2%)	2711 (29.6%)	68 (36.2%)	2354 (30.2%)	57 (38.3%)
**Chores**	None (Never)	782 (8.5%)	44 (9.6%)	586 (6.6%)	68 (3.7%)	629 (6.9%)	18 (9.1%)	549 (7.1%)	11 (7.5%)
	Low/Moderate Usage	4825 (52.2%)	250 (54.5%)	4595 (51.4%)	795 (43.2%)	4941 (54%)	100 (50.5%)	4104 (53.1%)	70 (47.9%)
	High (Everyday)	3631 (39.3%)	165 (35.9%)	3765 (42.1%)	976 (53.1%)	3582 (39.1%)	80 (40.4%)	3078 (39.8%)	65 (44.5%)
**Hang out with friends**	None (Never)	1971 (21%)	107 (23%)	2104 (23.4%)	298 (16.2%)	2043 (22.1%)	50 (25.6%)	1735 (22.3%)	29 (19.9%)
	Low/Moderate Usage	4797 (51.1%)	224 (48.2%)	5047 (56%)	1002 (54.5%)	5825 (63.1%)	123 (63.1%)	4796 (61.8%)	91 (62.3%)
	High (Everyday)	2628 (28%)	134 (28.8%)	1854 (20.6%)	538 (29.3%)	1360 (14.7%)	22 (11.3%)	1235 (15.9%)	26 (17.8%)
**Homework/Tutor**	None (Never)	1984 (21.1%)	92 (19.9%)	2557 (28.5%)	277 (15.1%)	1834 (19.7%)	44 (22.8%)	2075 (26.7%)	46 (30.9%)
	Low/Moderate Usage	4818 (51.2%)	227 (49.1%)	4611 (51.3%)	1009 (55%)	5803 (62.4%)	124 (64.2%)	4687 (60.3%)	88 (59.1%)
	High (Everyday)	2601 (27.7%)	143 (31%)	1815 (20.2%)	548 (29.9%)	1660 (17.9%)	25 (13%)	1015 (13.1%)	15 (10.1%)
**Arts**	None (Never)	3224 (33.9%)	159 (33.5%)	4127 (45.4%)	615 (33.3%)	5254 (56.5%)	113 (57.1%)	4959 (63.9%)	85 (57.8%)
	Low/Moderate Usage	5015 (52.8%)	249 (52.5%)	4214 (46.3%)	987 (53.5%)	3550 (38.2%)	69 (34.8%)	2428 (31.3%)	49 (33.3%)
	High (Everyday)	1261 (13.3%)	66 (13.9%)	753 (8.3%)	244 (13.2%)	489 (5.3%)	16 (8.1%)	379 (4.9%)	13 (8.8%)
**Music**	None (Never)	5878 (61.8%)	284 (60.7%)	6508 (71.5%)	1200 (65%)	6812 (73.2%)	149 (74.5%)	5805 (74.9%)	110 (74.3%)
	Low/Moderate Usage	2812 (29.6%)	144 (30.8%)	2060 (22.6%)	490 (26.5%)	2004 (21.5%)	45 (22.5%)	1520 (19.6%)	33 (22.3%)
	High (Everyday)	826 (8.7%)	40 (8.5%)	535 (5.9%)	157 (8.5%)	484 (5.2%)	6 (3%)	429 (5.5%)	5 (3.4%)

© 2025 Zhou M et al. *JAMA Network Open*.

**Organised sports**	None (Never)	2304 (24.4%)	114 (24.4%)	2789 (30.8%)	446 (24.3%)	3240 (34.6%)	72 (36%)	2994 (38.2%)	69 (46.3%)
	Low/Moderate Usage	5784 (61.1%)	283 (60.5%)	5235 (57.9%)	1082 (59%)	5360 (57.3%)	109 (54.5%)	4156 (53.1%)	68 (45.6%)
	High (Everyday)	1372 (14.5%)	71 (15.2%)	1021 (11.3%)	307 (16.7%)	760 (8.1%)	19 (9.5%)	683 (8.7%)	12 (8.1%)
**Read for fun**	None (Never)	1832 (19.9%)	99 (21.5%)	3084 (34.4%)	410 (22.3%)	4233 (47%)	97 (51.1%)	4112 (53%)	74 (50%)
	Low/Moderate Usage	4453 (48.3%)	199 (43.2%)	4123 (45.9%)	870 (47.3%)	3646 (40.5%)	69 (36.3%)	2784 (35.9%)	52 (35.1%)
	High (Everyday)	2933 (31.8%)	163 (35.4%)	1766 (19.7%)	558 (30.4%)	1123 (12.5%)	24 (12.6%)	864 (11.1%)	22 (14.9%)
**Young organization**	None (Never)	7998 (84.3%)	383 (81.1%)	8023 (88.5%)	1541 (83.6%)	8164 (88%)	167 (83.9%)	6850 (88.3%)	127 (87%)
	Low/Moderate Usage	1259 (13.3%)	69 (14.6%)	919 (10.1%)	267 (14.5%)	1017 (11%)	29 (14.6%)	819 (10.6%)	17 (11.6%)
	High (Everyday)	232 (2.4%)	20 (4.2%)	123 (1.4%)	36 (2%)	98 (1.1%)	3 (1.5%)	93 (1.2%)	2 (1.4%)

© 2025 Zhou M et al. *JAMA Network Open*.

eTable 4. Estimated Proportions in After-School Activity Frequency for Each Activity From 2019 to 2022

		Social media	TV	E-games	Chores	Hang out with friends	Homework/Tutor	Arts	Music	Organised sports	Read for fun	Young organisations
**Year**	**Frequency level**	Predicted proportion (95%CI)	Predicted proportion (95%CI)	Predicted proportion (95%CI)	Predicted proportion (95%CI)	Predicted proportion (95%CI)	Predicted proportion (95%CI)	Predicted proportion (95%CI)	Predicted proportion (95%CI)	Predicted proportion (95%CI)	Predicted proportion (95%CI)	Predicted proportion (95%CI)
2019	None (Never)	30.6% (29.1%, 32.1%)	1.8% (1.6%, 2.0%)	13.7% (12.8%, 14.6%)	2.8% (2.6%, 3.1%)	11.8% (11.1%, 12.5%)	13.7% (12.6%, 14.9%)	25.7% (24.5%, 27.0%)	70.3% (68.8%, 71.9%)	13.6% (12.7%, 14.7%)	10.8% (10.1%, 11.6%)	99.4% (99.3%, 99.6%)
2020		8.7% (8.1%, 9.4%)	1.4% (1.3%, 1.6%)	12.6% (11.8%, 13.5%)	2.3% (2.1%, 2.6%)	16.0% (15.2%, 16.9%)	20.8% (19.3%, 22.3%)	42.5% (41.0%, 44.1%)	82.0% (80.7%, 83.2%)	19.3% (18.1%, 20.7%)	26.0% (24.6%, 27.3%)	99.7% (99.6%, 99.8%)
2021		4.2% (3.8%, 4.7%)	1.9% (1.7%, 2.1%)	14.8% (13.7%, 15.9%)	2.7% (2.5%, 3.0%)	18.8% (17.7%, 19.9%)	21.2% (19.3%, 23.2%)	60.6% (58.9%, 62.2%)	84.2% (83.0%, 85.3%)	25.5% (23.6%, 27.4%)	43.5% (41.4%, 45.5%)	99.7% (99.6%, 99.8%)
2022		2.6% (2.3%, 2.9%)	1.8% (1.6%, 2.0%)	15.0% (13.8%, 16.2%)	2.7% (2.5%, 3.0%)	17.5% (16.4%, 18.7%)	30.0% (27.6%, 32.6%)	70.4% (68.8%, 72.0%)	85.4% (84.1%, 86.5%)	28.4% (26.3%, 30.5%)	52.6% (50.5%, 54.8%)	99.7% (99.6%, 99.8%)
2019	Low/Moderate Usage	43.5% (42.6%, 44.4%)	38.8% (37.4%, 40.2%)	66.1% (65.2%, 67.0%)	64.1% (62.8%, 65.5%)	70.5% (69.7%, 71.3%)	66.7% (65.8%, 67.5%)	68.7% (67.6%, 69.9%)	27.0% (25.6%, 28.3%)	81.3% (80.4%, 82.2%)	64.4% (63.4%, 65.4%)	0.5% (0.4%, 0.7%)
2020		29.6% (28.4%, 30.8%)	33.9% (32.5%, 35.3%)	65.7% (64.7%, 66.6%)	60.0% (58.6%, 61.4%)	70.9% (70.1%, 71.6%)	66.4% (65.5%, 67.2%)	54.8% (53.3%, 56.2%)	16.6% (15.5%, 17.7%)	77.3% (76.1%, 78.4%)	63.8% (62.7%, 64.9%)	0.3% (0.2%, 0.4%)
2021		18.0% (16.8%, 19.3%)	40.4% (38.7%, 42.0%)	66.4% (65.5%, 67.3%)	63.3% (61.9%, 64.7%)	70.2% (69.3%, 71.0%)	66.2% (65.2%, 67.2%)	38.1% (36.6%, 39.7%)	14.6% (13.6%, 15.7%)	72.1% (70.4%, 73.8%)	51.6% (49.9%, 53.3%)	0.3% (0.2%, 0.4%)
2022		12.0% (11.0%, 13.1%)	39.0% (37.2%, 40.7%)	66.5% (65.6%, 67.4%)	63.4% (61.9%, 64.9%)	70.5% (69.7%, 71.3%)	61.7% (60.0%, 63.4%)	28.7% (27.2%, 30.3%)	13.5% (12.5%, 14.6%)	69.6% (67.6%, 71.5%)	43.9% (42.0%, 45.8%)	0.3% (0.2%, 0.4%)
2019	High (Every day)	26.0% (24.6%, 27.3%)	59.5% (57.9%, 61.0%)	20.2% (19.0%, 21.4%)	33.0% (31.6%, 34.5%)	17.7% (16.8%, 18.7%)	19.6% (18.2%, 21.1%)	5.6% (5.2%, 6.0%)	2.7% (2.5%, 3.0%)	5.1% (4.6%, 5.6%)	24.7% (23.5%, 26.1%)	0.0% (0.0%, 0.0%)
2020		61.7% (60.0%, 63.3%)	64.7% (63.2%, 66.2%)	21.7% (20.5%, 23.0%)	37.7% (36.2%, 39.2%)	13.1% (12.4%, 13.9%)	12.9% (11.9%, 14.0%)	2.7% (2.5%, 2.9%)	1.4% (1.3%, 1.6%)	3.4% (3.1%, 3.7%)	10.2% (9.5%, 11.0%)	0.0% (0.0%, 0.0%)
2021		77.7% (76.0%, 79.3%)	57.7% (56.0%, 59.5%)	18.8% (17.5%, 20.2%)	33.9% (32.5%, 35.5%)	11.1% (10.3%, 11.9%)	12.6% (11.3%, 14.0%)	1.3% (1.2%, 1.5%)	1.2% (1.1%, 1.4%)	2.4% (2.2%, 2.7%)	4.9% (4.5%, 5.4%)	0.0% (0.0%, 0.0%)

© 2025 Zhou M et al. *JAMA Network Open*.

2022		85.4% (84.1%, 86.7%)	59.3% (57.4%, 61.1%)	18.5% (17.2%, 20.0%)	33.8% (32.2%, 35.4%)	12.0% (11.1%, 12.8%)	8.3% (7.4%, 9.3%)	0.9% (0.8%, 1.0%)	1.1% (1.0%, 1.3%)	2.1% (1.9%, 2.4%)	3.5% (3.1%, 3.8%)	0.0% (0.0%, 0.0%)

© 2025 Zhou M et al. *JAMA Network Open*.

eTable 5. Estimated Proportions in After-School Activity Frequency for Each Activity by Sex From 2019 to 2022

			Social media	TV	E-games	Chores	Hang out with friends	Homework/Tutor	Arts	Music	Organised sports	Read for fun	Young organisations
**Gender**	**Year**	**Frequency level**	Predicted proportion (95%CI)	Predicted proportion (95%CI)	Predicted proportion (95%CI)	Predicted proportion (95%CI)	Predicted proportion (95%CI)	Predicted proportion (95%CI)	Predicted proportion (95%CI)	Predicted proportion (95%CI)	Predicted proportion (95%CI)	Predicted proportion (95%CI)	Predicted proportion (95%CI)
Male	2019	None (Never)	39.3% (37.3%, 41.3%)	2.2% (2.0%, 2.5%)	3.9% (3.5%, 4.4%)	3.8% (3.4%, 4.2%)	11.4% (10.5%, 12.4%)	15.2% (13.9%, 16.6%)	46.7% (44.6%, 48.9%)	78.7% (76.7%, 80.6%)	13.7% (12.5%, 15.1%)	15.0% (13.8%, 16.2%)	96.1% (12.5%, 15.1%)
	2020		13.3% (12.2%, 14.4%)	1.8% (1.6%, 2.0%)	3.2% (2.9%, 3.6%)	3.1% (2.8%, 3.5%)	15.6% (14.4%, 16.8%)	23.5% (21.7%, 25.3%)	64.1% (62.0%, 66.1%)	86.7% (85.1%, 88.1%)	19.6% (18.0%, 21.3%)	33.2% (31.2%, 35.2%)	97.6% (18.0%, 21.3%)
	2021		7.0% (6.3%, 7.8%)	2.5% (2.2%, 2.8%)	4.0% (3.5%, 4.5%)	3.4% (3.0%, 3.8%)	18.3% (16.9%, 19.7%)	23.4% (21.1%, 25.8%)	78.2% (76.5%, 79.9%)	87.1% (85.6%, 88.5%)	26.1% (23.9%, 28.4%)	54.5% (52.0%, 56.9%)	97.3% (23.9%, 28.4%)
	2022		4.3% (3.8%, 4.9%)	2.5% (2.2%, 2.9%)	3.4% (3.0%, 3.9%)	3.6% (3.1%, 4.0%)	17.1% (15.8%, 18.6%)	32.9% (30.0%, 35.9%)	85.7% (84.2%, 87.1%)	88.0% (86.5%, 89.4%)	26.4% (24.1%, 28.8%)	68.0% (65.6%, 70.3%)	97.4% (24.1%, 28.8%)
	2019	Low/Moderate Usage	40.2% (38.9%, 41.5%)	35.1% (33.4%, 36.9%)	56.8% (54.8%, 58.8%)	66.9% (65.1%, 68.6%)	69.3% (68.1%, 70.4%)	64.8% (63.7%, 65.9%)	49.4% (47.5%, 51.4%)	19.4% (17.8%, 21.2%)	78.6% (77.4%, 79.7%)	64.1% (62.7%, 65.4%)	3.7% (77.4%, 79.7%)
	2020		34.5% (33.1%, 35.9%)	30.1% (28.4%, 31.8%)	52.5% (50.4%, 54.7%)	63.4% (61.5%, 65.2%)	70.1% (69.0%, 71.2%)	63.8% (62.6%, 65.1%)	34.0% (32.1%, 36.0%)	12.3% (11.0%, 13.7%)	75.3% (73.8%, 76.7%)	58.2% (56.6%, 59.8%)	2.2% (73.8%, 76.7%)
	2021		24.0% (22.5%, 25.7%)	37.2% (35.4%, 39.0%)	57.0% (54.7%, 59.3%)	64.7% (62.9%, 66.6%)	69.6% (68.5%, 70.7%)	63.9% (62.5%, 65.2%)	20.8% (19.3%, 22.4%)	11.9% (10.7%, 13.2%)	70.3% (68.3%, 72.2%)	41.8% (39.7%, 43.9%)	2.6% (68.3%, 72.2%)
	2022		16.9% (15.5%, 18.5%)	37.8% (35.8%, 39.8%)	53.9% (51.4%, 56.4%)	65.9% (63.9%, 67.7%)	69.9% (68.8%, 71.0%)	58.8% (56.7%, 60.8%)	13.7% (12.4%, 15.1%)	11.0% (9.8%, 12.4%)	70.0% (67.9%, 72.1%)	29.9% (27.8%, 32.1%)	2.5% (67.9%, 72.1%)
	2019	High (Every day)	20.5% (19.1%, 22.0%)	62.7% (60.7%, 64.6%)	39.3% (37.0%, 41.6%)	29.3% (27.5%, 31.3%)	19.3% (18.0%, 20.7%)	20.0% (18.4%, 21.7%)	3.9% (3.4%, 4.3%)	1.8% (1.6%, 2.2%)	7.7% (6.9%, 8.5%)	21.0% (19.5%, 22.6%)	0.3% (6.9%, 8.5%)
	2020		52.2% (50.1%, 54.3%)	68.2% (66.3%, 70.0%)	44.2% (41.9%, 46.6%)	33.5% (31.5%, 35.6%)	14.3% (13.3%, 15.5%)	12.7% (11.6%, 14.0%)	1.9% (1.7%, 2.2%)	1.1% (0.9%, 1.3%)	5.2% (4.6%, 5.8%)	8.6% (7.8%, 9.5%)	0.2% (4.6%, 5.8%)
	2021		69.0% (66.7%, 71.2%)	60.4% (58.3%, 62.4%)	39.0% (36.4%, 41.7%)	31.9% (29.9%, 34.0%)	12.1% (11.1%, 13.2%)	12.8% (11.4%, 14.4%)	1.0% (0.8%, 1.1%)	1.0% (0.9%, 1.2%)	3.6% (3.2%, 4.1%)	3.8% (3.3%, 4.2%)	0.2% (3.2%, 4.1%)

© 2025 Zhou M et al. *JAMA Network Open*.

	2022		78.7% (76.7%, 80.6%)	59.7% (57.5%, 61.8%)	42.6% (39.8%, 45.5%)	30.6% (28.5%, 32.8%)	13.0% (11.9%, 14.2%)	8.4% (7.3%, 9.5%)	0.6% (0.5%, 0.7%)	0.9% (0.8%, 1.1%)	3.6% (3.1%, 4.1%)	2.2% (1.9%, 2.5%)	0.2% (3.1%, 4.1%)
Female	2019	None (Never)	22.3% (20.6%, 24.0%)	1.3% (1.2%, 1.6%)	34.6% (32.6%, 36.7%)	2.1% (1.8%, 2.4%)	12.3% (11.3%, 13.3%)	11.9% (11.0%, 13.0%)	10.8% (9.9%, 11.9%)	61.7% (59.4%, 64.0%)	12.9% (11.5%, 14.3%)	7.4% (6.7%, 8.2%)	99.7% (99.6%, 99.8%)
	2020		5.3% (4.7%, 5.9%)	1.1% (0.9%, 1.3%)	34.8% (32.8%, 36.9%)	1.7% (1.5%, 1.9%)	16.6% (15.4%, 17.9%)	17.7% (16.0%, 19.0%)	21.7% (20.1%, 23.4%)	77.1% (75.1%, 78.9%)	18.4% (16.7%, 20.3%)	19.3% (17.8%, 20.8%)	99.9% (99.8%, 99.9%)
	2021		2.1% (1.8%, 2.4%)	1.4% (1.2%, 1.6%)	38.5% (36.2%, 40.9%)	2.2% (1.9%, 2.5%)	19.0% (17.5%, 20.5%)	16.9% (15.0%, 19.0%)	37.3% (35.0%, 39.7%)	81.2% (79.3%, 82.9%)	24.5% (22.1%, 27.0%)	34.2% (31.8%, 36.6%)	99.9% (99.8%, 99.9%)
	2022		1.2% (1.0%, 1.5%)	1.2% (1.0%, 1.4%)	42.6% (40.1%, 45.2%)	2.1% (1.8%, 2.4%)	17.6% (16.2%, 19.1%)	24.3% (22.0%, 27.0%)	46.9% (44.2%, 49.6%)	82.7% (80.8%, 84.5%)	30.6% (27.8%, 33.7%)	38.6% (35.9%, 41.3%)	99.9% (99.8%, 99.9%)
	2019	Low/Moderate Usage	45.3% (43.8%, 46.8%)	43.0% (41.0%, 45.1%)	55.8% (54.2%, 57.4%)	60.8% (58.8%, 62.8%)	71.6% (70.5%, 72.7%)	68.6% (67.0%, 70.0%)	79.2% (78.1%, 80.3%)	34.6% (32.6%, 36.6%)	84.2% (82.8%, 85.4%)	62.8% (61.2%, 64.4%)	0.3% (0.2%, 0.4%)
	2020		23.5% (22.0%, 25.2%)	38.3% (36.2%, 40.4%)	55.7% (54.0%, 57.3%)	56.1% (54.0%, 58.2%)	71.5% (70.4%, 72.6%)	69.1% (68.0%, 70.0%)	73.7% (72.2%, 75.2%)	21.1% (19.5%, 22.9%)	79.6% (77.8%, 81.3%)	68.3% (67.0%, 69.5%)	0.1% (0.1%, 0.2%)
	2021		11.3% (10.1%, 12.6%)	43.8% (41.4%, 46.2%)	53.2% (51.4%, 55.1%)	61.9% (59.8%, 63.9%)	70.8% (69.6%, 71.9%)	69.2% (68.0%, 70.0%)	60.5% (58.3%, 62.6%)	17.4% (15.8%, 19.1%)	74.2% (71.7%, 76.5%)	59.7% (57.7%, 61.7%)	0.1% (0.1%, 0.2%)
	2022		7.1% (6.2%, 8.1%)	40.2% (37.7%, 42.7%)	50.3% (48.3%, 52.4%)	60.7% (58.5%, 62.9%)	71.2% (70.1%, 72.4%)	66.5% (65.0%, 68.0%)	51.6% (49.1%, 54.1%)	16.0% (14.4%, 17.7%)	68.3% (65.4%, 71.1%)	56.3% (54.0%, 58.5%)	0.1% (0.1%, 0.2%)
	2019	High (Every day)	32.4% (30.4%, 34.5%)	55.6% (53.4%, 57.8%)	9.6% (8.7%, 10.5%)	37.1% (35.0%, 39.2%)	16.1% (14.9%, 17.3%)	19.5% (18.0%, 21.0%)	10.0% (9.1%, 10.9%)	3.7% (3.3%, 4.3%)	3.0% (2.6%, 3.5%)	29.8% (27.8%, 31.8%)	0.0% (0.0%, 0.0%)
	2020		71.2% (69.1%, 73.2%)	60.6% (58.4%, 62.8%)	9.5% (8.7%, 10.5%)	42.2% (40.0%, 44.5%)	11.9% (10.9%, 12.9%)	13.3% (12.0%, 15.0%)	4.6% (4.1%, 5.2%)	1.8% (1.6%, 2.1%)	2.0% (1.7%, 2.3%)	12.5% (11.4%, 13.7%)	0.0% (0.0%, 0.0%)
	2021		86.6% (85.1%, 88.1%)	54.8% (52.3%, 57.3%)	8.2% (7.4%, 9.1%)	36.0% (33.9%, 38.2%)	10.3% (9.4%, 11.3%)	13.9% (12.0%, 16.0%)	2.2% (1.9%, 2.5%)	1.4% (1.2%, 1.7%)	1.4% (1.2%, 1.6%)	6.2% (5.5%, 6.9%)	0.0% (0.0%, 0.0%)
	2022		91.7% (90.5%, 92.8%)	58.6% (56.0%, 61.2%)	7.0% (6.3%, 7.9%)	37.3% (34.9%, 39.6%)	11.2% (10.1%, 12.3%)	9.3% (8.0%, 11.0%)	1.5% (1.3%, 1.7%)	1.3% (1.1%, 1.5%)	1.0% (0.9%, 1.2%)	5.1% (4.5%, 5.8%)	0.0% (0.0%, 0.0%)

© 2025 Zhou M et al. *JAMA Network Open*.

eFigure. Participant Flowchart

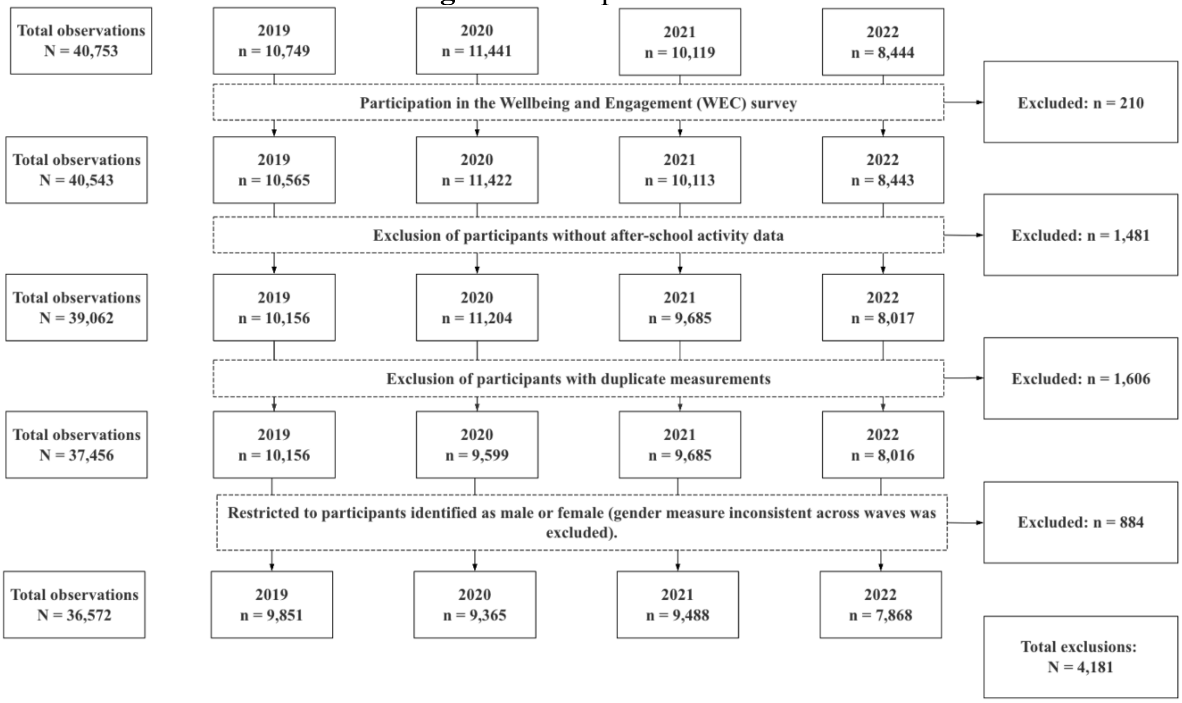

© 2025 Zhou M et al. *JAMA Network Open*.
