# Supplementary material for: Postpandemic After-School Activities Among Youths in Australia
Source: JAMA Netw Open. 2025 Nov 14;8(11):e2543637. doi: 10.1001/jamanetworkopen.2025.43637 (PMC12619106; doi:10.1001/jamanetworkopen.2025.43637)
Supplement: Supplement 2. — Data Sharing Statement [file jamanetwopen-e2543637-s002.pdf]

Data Sharing Statement

Zhou. Postpandemic After-School Activities Among Youths in Australia. *JAMA Netw Open*. Published November 14, 2025. doi:10.1001/jamanetworkopen.2025.43637

Data
Data available: No
